# Validation of WHO 2017 Classification and Identification of Prognostic Factors in Patients with Pancreatic Neuroendocrine Neoplasms: A Real-World Experience in Taiwan

**DOI:** 10.7150/jca.123900

**Published:** 2025-10-27

**Authors:** Wei-Pang Ho, Wen-Chi Chou, Chia-Hsun Hsieh, Ming-Mo Hou, Wen-chi Shen, Pei-Wei Huang, Chiao-En Wu, Chih-Chung Hsu, Wen-Cheng Chang, Yung-Chia Kuo, Hung-Chih Hsu, Ching-Fu Chang, Po-Jung Su, Ren-Chin Wu, Jen-Shi Chen, Wen-Kuan Huang

**Affiliations:** 1 Division of Hematology-Oncology, Department of Internal Medicine, Chang Gung Memorial Hospital at Linkou, Taoyuan, Taiwan.; 2 College of Medicine, Chang Gung University, Taoyuan, Taiwan.; 3 Department of Pathology, Chang Gung Memorial Hospital at Linkou, Taoyuan, Taiwan.

**Keywords:** pancreatic neuroendocrine tumor, neuroendocrine carcinoma, WHO 2017 classification, prognostic factors

## Abstract

**Background:** Pancreatic neuroendocrine tumors (PanNETs) are rare neoplasms with an increasing incidence. This study aims to validate the clinical relevance of the WHO 2017 classification system in the Taiwanese population and identify independent prognostic factors for patients with PanNETs.

**Materials and methods:** We conducted a retrospective analysis of 176 patients with PanNETs from the Chang Gung Medical Hospital at Linkou in Taiwan, spanning the years 2009 to 2022. Pathology reports were reassessed according to the WHO 2017 classification. Clinical characteristics, treatment patterns, and survival outcomes were documented, with subgroup analyses to compare grade 3 (G3) neuroendocrine tumors and neuroendocrine carcinomas (NEC).

**Results:** The overall 5-year survival rate was 58.7%, with median survival of 107.6 months. Survival rates showed clear stratification across WHO 2017 classifications: G1 (83.1%, median 141.0 months), G2 (55.0%, median 105.2 months), G3 (14.6%, median 21.5 months), and NEC (9.4%, median 19.6 months). Multivariate analysis identified five independent prognostic factors: age over 60 years (HR 1.70), tumor size >2cm (HR 1.893), lymph node involvement (HR 1.801), distant metastasis (HR 3.042), and NEC classification (HR 2.382). NEC demonstrated significantly higher lymph node involvement (81% vs 48%, p=0.026), higher Ki-67 index (69 vs 43.8, p<0.001), and higher rates of metastases compared with G3 NET.

**Conclusions:** Our findings validate the prognostic utility of the WHO 2017 classification, particularly in differentiating NET G3 from NEC. This refined classification system, combined with identified prognostic factors, provides valuable guidance for clinical decision-making and treatment selection in patients with PanNETs.

## Introduction

Pancreatic neuroendocrine tumor (PanNETs) are rare neoplasms, accounting for ≤1% of all pancreatic tumors 1, 2. Significantly, recent years have seen a notable increase in the incidence of pancreatic neuroendocrine tumors 2, 3, a phenomenon that can largely be attributed to advancements in diagnostic imaging and techniques 4. A previous epidemiological study conducted in Taiwan reported an incidence of 0.446 cases per 100,000 individuals diagnosed with neuroendocrine tumors, further emphasizing the growing incidence 5. This rise underscores the necessity for a comprehensive understanding of PanNETs to improve their effective management.

PanNETs represent a heterogeneous group of neoplasms with diverse clinical manifestations, pathological characteristics, and long-term prognoses 6-8. Notably, the updated World Health Organization (WHO) 2017 classification has provided a more refined framework for categorizing PanNETs, particularly by distinguishing well-differentiated Grade 3 (G3) neuroendocrine tumors (NET) from neuroendocrine carcinomas (NEC) 9-11, with significant implications for prognosis. The evolution of classification systems, particularly the transition from the WHO 2010 classification to the updated WHO 2017 classification, has significantly refined the framework for PanNETs categorization. The updated classification distinguishes well-differentiated Grade 3 (G3) neuroendocrine tumors from NEC, providing clearer differentiation between PanNETs subtypes 12 This enhanced clarity has critical implications for treatment strategies and prognostic assessments, enabling clinicians to better tailor interventions to the specific tumor characteristics of each patient 13.

To validate the clinical relevance and prognostic value of the WHO 2017 classification system in the Taiwanese population with PanNETs, we conducted a comprehensive retrospective analysis at Chang Gung Medical Hospital at Linkou, the largest medical center in Taiwan. Second, to identify independent prognostic factors that could guide clinical decision-making and treatment planning. Furthermore, this study seeks to identify key clinical and pathological prognostic factors that influence survival outcomes in patients with PanNETs. Through this analysis, we aimed to provide evidence-based insights that would enhance the management of patients with PanNETs in Taiwan and contribute to the broader understanding of these tumors in Asian populations.

## Materials and Methods

A comprehensive retrospective analysis was conducted on 176 cases of PanNETs collected from Chang Gung Memorial Hospital at Linkou, Taiwan, encompassing data from January 2009 to December 2022. Patient included in this study had a prior histopathological diagnosis of PanNETs. Clinical characteristics, symptom presentation, and overall survival outcomes were comprehensively documented for all cases.

To ensure diagnostic precision and appropriate staging, pathology reports for all cases were meticulously re-evaluated by expert pathologists according to both the WHO 2010 and updated WHO 2017 classifications. Patients were classified into four groups based on the WHO 2017 classification: Grade 1 (G1), Grade 2 (G2), Grade 3 (G3), and NEC.

Survival outcomes, including median survival time and five-year survival rates, were calculated for the entire cohort and specific PanNETs subgroups. Kaplan-Meier survival curves were used for initial survival analyses, with subgroup stratification based on the WHO 2017 classification to explore survival trends. To identify independent prognostic factors, both univariate and multivariate Cox regression analyses were performed. Variables analyzed included age (≤60 vs >60 years), presence or absence of symptoms at diagnosis, tumor size (≤2cm vs >2cm), lymph node involvement, distant metastasis, Ki-67 index (≤55% vs >55%), and WHO 2017 classification groups. Statistical significance was set at a p-value of 0.05.

To investigate the potential differences in tumor biology, a subgroup analysis was performed to compare the G3 NET and NEC groups, focusing on tumor characteristics, Ki-67 index distribution, treatment patterns, and oncologic outcomes. Also, we utilized Chi-square analysis to investigate the relationship between KI-67 expression and high-grade pancreatic neuroendocrine neoplasms (NET G3 and NEC). Furthermore, we performed a subgroup analysis of high-grade neuroendocrine neoplasms based on differing levels of Ki-67 expression.

All statistical analyses were performed using SPSS version 26 software. Categorical variables were compared using chi-square or Fisher's exact tests as appropriate, while continuous variables were analyzed using Student's t-test or Mann-Whitney U test based on their distribution. This comprehensive analysis provided insights into the clinical characteristics, prognostic factors, and treatment outcomes across different PanNETs subgroups, particularly emphasizing the distinctions between G3 and NEC categories.

## Results

According to the updated WHO 2017 classification, PanNETs could be classified into four groups, including Grade 1 (G1), Grade 2 (G2), Grade 3 (G3) and NEC. Table 1indicates that patients in the G3 (61.4 years) and NEC (64.1 years) groups were generally older than those in the G1 (57.8 years) and G2 (52.1 years) groups. These higher-grade groups (G3 and NEC) also exhibited more frequent symptoms, particularly abdominal pain (G3: 64.0%, NEC: 62.5%), jaundice (G3: 8.0%, NEC: 12.5%), and poor appetite (G3: 12.0%, NEC: 25.0%). In contrast, approximately one-third of G1 (33.3%) and G2 (29.8%) patients were asymptomatic and diagnosed incidentally. The most common symptoms in PanNETs are abdominal pain and hypo/hyperglycemia (Table 1). A greater proportion of G1 patients underwent surgery following the incidental discovery of PanNETs. Patients in the NEC group displayed the highest rates of distant metastases, lymph node involvement, and elevated Ki-67 indices. Additionally, this group had the largest proportion of patients receiving systemic therapies such as chemotherapy.

**Figure 1:**
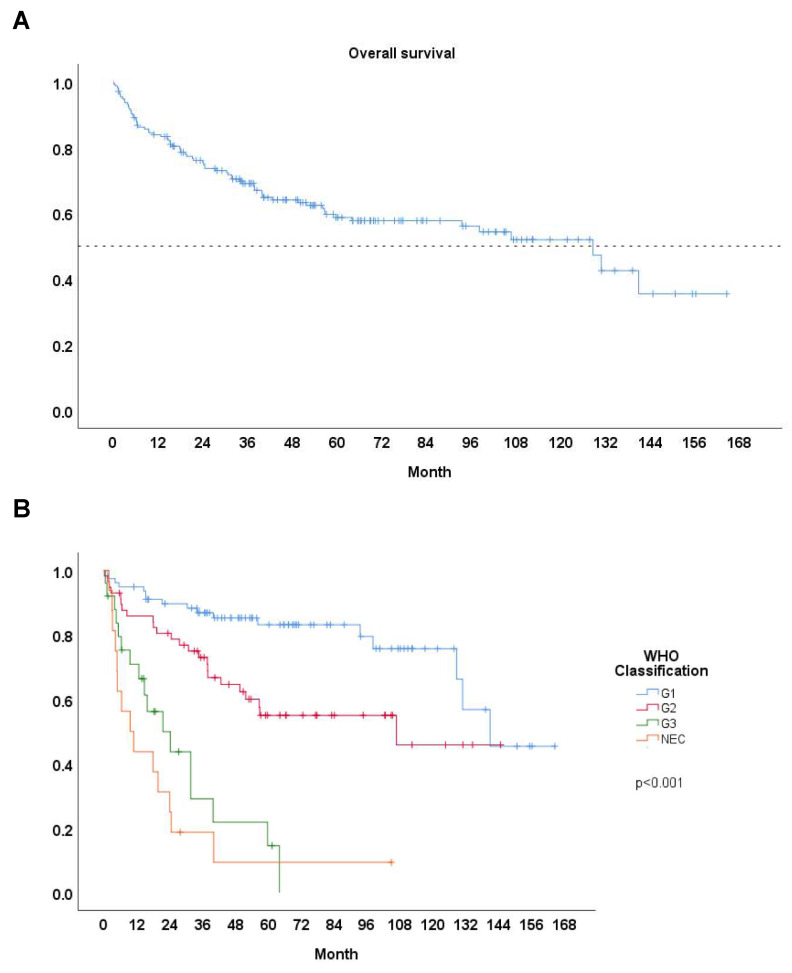
**A** Survival curve: All. **B** Survival curve: Based on WHO 2017 classification.

We documented the median survival time and 5-year survival rates to compare different groups of patients with PanNETs. Among the entire cohort of 176 patients, the 5-year survival rate was 58.7%, with a median survival time of 107.6 months (Fig. 1A). Patients with G1 tumors showed the highest survival rates, with a 5-year survival of 83.1% (n = 78) and a median survival time of 141.0 months (Table 2). G2 patients had a 5-year survival rate of 55.0% (n = 57) and a median survival time of 105.2 months. In contrast, G3 patients had poorer outcomes, with a 5-year survival rate of 14.6% (n = 25) and a median survival time of 21.5 months. Patients with NEC exhibited the worst survival outcomes, with a 5-year survival rate of 9.4% (n = 16) and a median survival time of 19.6 months. As highlighted by the updated WHO 2017 classification, the G3 group shows better survival outcomes compared to NEC group, but poorer outcomes compared than G2 group, as confirmed by our findings (Fig. 1B).

To investigate the prognostic factors, we conducted both univariate and multivariate analyses (Table 3). The univariate analysis identified several significant prognostic factors, including age over 60 years, absence of symptoms at diagnosis, tumor size > 2cm, lymph node involvement, distant metastasis, Ki-67 index > 55%, and NEC classification under WHO 2017 classification (all p < 0.05). In multivariate analysis, age over 60 years (HR 1.70, p=0.013), tumor size > 2cm (HR 1.893, p = 0.042), lymph node involvement (HR 1.801, p = 0.042), distant metastasis (HR 3.042, p = 0.001), and NEC classification (HR 2.382, p = 0.016) remained independently significant.

The comparison between NET G3 (N = 25) and NEC (N = 16) reveals some remarkable differences in their characteristics and treatment patterns. While tumor size was comparable (5.51 vs 5.07, p = 0.645), NEC showed significantly higher lymph node involvement (81% vs 48%, p = 0.026) and Ki-67 proliferation index (69 vs 43.8, p < 0.001) (Table 4). Table 5 further underscores the distinction in Ki-67 distribution, with NET G3 cases predominantly exhibiting Ki-67 < 55% (18 vs. 2 in NEC) and NEC cases showing Ki-67 > 55% (14 vs. 7 in NET G3), yielding a robust statistical association (p < 0.001). Treatment approaches differed markedly, with NEC patients receiving more chemotherapy overall (88% vs 68%), particularly Etoposide + Cisplatin (69% vs 32%). Notably, NET G3 patients had access to more diverse treatment options, including targeted therapy with Sunitinib (8%) and somatostatin analog therapy (12%), which were not utilized in NEC patients. Notably, NEC patients had a higher objective response rate (29% vs. 19%, p = 0.547), although disease control rate and progression-free survival did not differ significantly.

In our study, Chi-square analysis indicated that a high KI-67 group (cut-off point: KI-67 ≥ 55%) is more prevalent in NEC, with a significance level of P < 0.001 (Table 5). In the subgroup analysis of high-grade neuroendocrine neoplasms, no significant differences were noted in tumor size, lymph node involvement, distant metastasis, or response rate. However, the PFS was significantly longer in patients receiving chemotherapy as first-line systemic therapy, with a PFS of 4.87 months compared to 3.40 months (p = 0.044). The overall survival was also noteworthy, with values of 33.83 months for the low KI-67 group compared to 10.83 months for the high KI-67 group (p = 0.001).

## Discussion

Our analysis demonstrated clear prognostic stratification across these groups, with 5-year survival rates ranging from 83.1% in G1 tumors to just 9.4% in NEC cases. The validity of the WHO 2017 classification was particularly evident in distinguishing between G3 and NEC categories, which showed distinct clinical behaviors despite both being high-grade tumors. Our analysis revealed biological and clinical differences between these high-grade categories: NET G3 and NEC demonstrated distinct Ki-67 distribution patterns and significantly different lymph node involvement. Notably, treatment responses and options also differed markedly between these groups, with NET G3 patients having access to more diverse therapeutic options including targeted therapy and somatostatin analogs, while NEC patients primarily received chemotherapy regimens. These characterization of high-grade PanNETs provides evidence supporting the clinical utility of the WHO 2017 classification and suggests that these categories may benefit from distinct therapeutic approaches.

Subtypes of PanNETs show distinct biological behaviors, ranging from indolent tumors with slow growth and minimal symptoms to aggressive high-grade variants like NEC 14. Our study reinforces these differences, highlighting the importance of effective classification in clinical practice. Prior research has shown that the updated WHO 2017 classification is more accurate than the WHO 2010 system 15. Despite a relatively small sample size, our findings support the efficacy of WHO 2017 classification in categorizing PanNETs.

Previous studies have established different molecular mechanisms and genetic backgrounds between G3 and NEC 16. Based on prior data, the most prevalent mutations in pancreatic neuroendocrine tumors include those in MEN1 (44%), DAXX/ATRX (43%) 17, 18, and genes related to the mTOR pathway. In contrast, the most commonly mutated genes in NEC are TP53 and RB, with NECs exhibiting significantly higher mutation burden 19, 20. Notably, G3 patients demonstrated a significantly different Ki-67 distribution pattern compared to NEC (p<0.001), with the majority of G3 cases (18/25, 72%) showing Ki-67 indices below 55%, while most NEC cases (14/16, 87.5%) exhibited Ki-67 indices above 55%. These findings underscore the higher proliferative activity in NECs, consistent with their aggressive clinical behavior. Regarding prognosis, our findings suggest that the G3 group may display a more favorable prognosis than previously anticipated, particularly when compared to the NEC group. A deeper understanding of these clinical and pathological factors is essential for optimizing treatment strategies and enhancing patient management in this population.

Systemic treatment for PanNETs includes various modalities such as cytotoxic chemotherapy, somatostatin analogs, and targeted therapies 21. However, it is important to note that there is currently no established standard chemotherapy specifically for this disease, and the response to chemotherapy can vary depending on the tumor grade. The first-line therapy typically consists of a regimen comprising platinum-based agents in combination with etoposide in NEC. Clinical studies have reported response rates for this treatment combination ranging from 31% to 67 22. Well-differentiated G3 tumors typically exhibit slow proliferation and tend to be resistant to most chemotherapeutic agents 23. They have a limited response to platinum-based chemotherapy, so other treatments may be considered, such as temozolomide-based chemotherapy or peptide receptor radiotherapy. In Table 4, we attempted to compare the impact of chemotherapy between G3 and NEC groups. No statistically significant differences were noted in response rate and PFS between the NEC and G3 groups receiving first line chemotherapy. Also, previous data revealed, as KI-67 elevation, response rate increased and survival outcome decreased 24. We conducted a further investigation into the role of KI-67 in high-grade PanNETs. The low KI-67 group exhibited better OS and PFS. Despite achieving a higher objective response rate (ORR) to initial chemotherapy, the high Ki-67 group is characterized by more aggressive tumor behavior, including a higher prevalence of NEC and distant metastasis. These findings underscore Ki-67 as a critical prognostic factor, with high expression indicating poor long-term despite initial treatment efficacy.

This study has several limitations that should be considered. First, its retrospective design involved analyzing data and recruiting patients over an extended period. Second, the use of Kaplan-Meier methods to estimate cumulative overall survival may be affected by cases with limited follow-up. Additionally, the analysis was confined to a single medical center in Taiwan, potentially limiting statistical power for assessing various factors and survival outcomes. Therefore, a prospectively designed multicenter study with longer follow-up is essential to validate these findings.

## Conclusion

Our retrospective analysis highlights the complex interplay between prognostic factors and overall survival in patients with PanNETs, revealing key indicators that significantly influence outcomes and enabling more tailored clinical management. Additionally, the updated WHO 2017 classification has proven pivotal in distinguishing G3 PanNETs from NEC, enhancing patient stratification based on tumor biology and guiding the selection of appropriate treatments. Integrating this refined classification and these key indicators into clinical practice holds substantial potential for improving prognostic assessments and optimizing treatment strategies for high-grade PanNETs.

## Figures and Tables

**Table 1:** Subgroup of The WHO 2017 classification of the neuroendocrine neoplasm of the pancreases

	Well differentiated			Poor differentiated
Subgroup	G1(N=78)	G2(N=57)	G3(N=25)	NEC(N=16)
Characteristic				
Age	57.8(16-84)	52.1(16-73)	61.4(24-85)	64.1(45-81)
Male	47.4 %	57.8 %	60.0 %	68.8 %
Female	52.6 %	42.2 %	40.0 %	31.3 %
Symptoms				
Abdominal pain	37.2 %	47.4 %	64.0 %	62.5 %
Body weight loss	3.8 %	5.3 %	20.0 %	12.5 %
Diahhrea	3.8 %	5.3 %	0.0 %	12.5 %
Gastrointestinal bleeding	5.1 %	3.5 %	4.0 %	0.0 %
Hypo/Hyperglycemia	14.1 %	17.5 %	0.0 %	0.0 %
jaundice	0.0 %	0.0 %	8.0 %	12.5 %
Poor appetite	3.8 %	5.3 %	12.0 %	25.0 %
No symptoms	33.3 %	29.8 %	16.0 %	0.0 %
AJCC				
Stage1	50.0 %	17.5 %	0.0 %	0.0 %
Stage2	26.9 %	26.3 %	4.0 %	0.0 %
Stage3	6.4 %	7.0 %	12.0 %	12.5 %
Stage4	16.7 %	49.1 %	84.0 %	87.5 %
Treatment				
Surgery	82.1 %	63.2 %	32.0 %	18.8 %
Somatostatin	16.7 %	14.0 %	28.0 %	12.5 %
Chemotherapy	1.3 %	21.1 %	68.0 %	87.5 %
Targeted therapy	11.5 %	50.9 %	16.0 %	12.5 %

NEC: Neuroendocrine carcinoma.

**Table 2:** Median survival time and 5-year survival

	MST	5-year survival
G1	141.0	83.1%
G2	105.2	55.0%
G3	21.5	14.6%
NEC	19.6	9.4%
ALL	107.6	58.7%

P value <0.001 Note. MST: Median survival time (month). NEC: Neuroendocrine carcinoma.

**Table 3:** The univariate analysis & multivariate analysis based on WHO 2017 and 2010 criteria of PNET

	Univariate Analysis HR(CI), P value	Multivariate Analysis HR(CI), P value
Age, year		
< 60		
> 60	1.666(1.044-2.658), 0.032	1.70(1.006-2.890), 0.013
Sex		
Female		
Male	1.161(0.728-1.851), 0.530	
Carcinoid symptoms		
No		
Yes	1.301(0.519-3.259), 0.588	
Hypo/hyperglycemia		
No		
Yes	0.814(0.259-2.593), 0.720	
No symptom		
No		
Yes	0.499(0.268-0.928), 0.018	0.759(0.404-1.427), 0.392
CgA > 10 X upper limit		
No		
Yes	1.944(1.039-3.637), 0.053	
SSTR positive		
No		
Yes	0.805(0.399-1.623), 0.534	
Size > 2cm		
No		
Yes	3.233(1.800-5.806), <0.001	1.893(1.024-3.500), 0.042
Lymph node involvement		
No		
Yes	4.324(2.693-6.491), <0.001	1.801 (1.021-3.177), 0.042
Distant metastasis		
No		
Yes	5.316(3.140-8.999), <0.001	3.042(1.618-5.718), 0.001
Ki-67 >55		
No		
Yes	2.060(1.250-3.396), 0.007	
WHO2017		
NET G1/G2/G3		
NEC	5.298(2.892-9.703), <0.001	2.382(1.178-4.817), 0.016

NET: Neuroendocrine tumor; NEC: Neuroendocrine carcinoma.

**Table 4:** The comparison between NET G3 and NEC

	NET G3 (N=25)	NEC (N=16)	P value
Characteristics			
Tumor size	5.51	5.07	0.645
Lymph node involvement	48%	81%	0.026
Distant metastasis	84%	88%	0.760
Mitotic index	12.20	15.60	0.428
KI-67	43.83	69.06	<0.000
1L systemic treatment			
Chemotherapy	68%	88%	
Etoposide + Cisplatin	32%	69%	
Etoposide + Carboplatin	4%	6%	
Dacarbazine only	24%	0%	
Dacarbazine + 5FU	4%	0%	
Gemcitabine + TS-1	0%	6%	
Gemcitabine + Nab-Paclitaxel	0%	6%	
Targeted therapy	8%	0%	
Sunitinib	8%	0%	
Somatostatin analog combined	12%	0%	
No systemic treatment	20%	13%	
After 1L chemotherapy treatment (image follow up in 3 months)			
Objective response rate	19%	29%	0.547
Disease control rate	44%	43%	0.962
Progression-free survival(month)	5.36	3.40	0.743

**Table 5:** Characteristics of high-grade PanNENs stratified by Ki-67 (<55% vs ≥55%)

	High KI group (N = 15)	Low KI group (N = 16)	P
High-grade Neuroendocrine neoplasm	N (%)	N (%)	< 0.001
NET G3	3 (20%)	14 (88%)	
NEC	12 (80%)	2 (13%)	
Characteristics			
Tumor size	5.79	0.655	0.655
Lymph node involvement	73%	56%	0.335
Distant metastasis	80%	88%	0.588
1L systemic treatment			
Etoposide + Cisplatin	80%	43.7%	
Etoposide + Carboplatin	6.7%	6.3%	
Dacarbazine only	0%	43.7%	
Dacarbazine + 5FU	0%	6.3%	
Gemcitabine + TS-1	6.7%	0%	
Gemcitabine + Nab-Paclitaxel	6.7%	0%	
After 1L chemotherapy treatment (image follow up in 3 month)			
Objective response rate	27%	19%	0.614
Disease control rate	33%	50%	0.363
Progression-free survival(month)	3.40	4.87	0.044*
Overall survival(month)	10.83	33.83	0.001*

NET: Neuroendocrine tumor; NEC: Neuroendocrine carcinoma.
